# Enhanced Surveillance of >1100 Pesticides and Natural Toxins in Food: Harnessing the Capabilities of LC-HRMS for Reliable Identification and Quantification

**DOI:** 10.3390/foods13193040

**Published:** 2024-09-25

**Authors:** Thomas Bessaire, Marie-Claude Savoy, Marion Ernest, Nicolas Christinat, Flavia Badoud, Aurélien Desmarchelier, Benoit Carrères, Wai-Chinn Chan, Xiaoyu Wang, Thierry Delatour

**Affiliations:** 1 Nestlé Research Center, Vers-chez-les-Blanc, CH-1000 Lausanne, Switzerland; marieclaude.savoy@rd.nestle.com (M.-C.S.); marion.ernest@rdls.nestle.com (M.E.); nicolas.christinat@rd.nestle.com (N.C.); flavia.nagy@rd.nestle.com (F.B.); aurelien.desmarchelier@rdls.nestle.com (A.D.); benoit.carreres@rd.nestle.com (B.C.); thierry.delatour@rdls.nestle.com (T.D.); 2 Nestlé Quality Assurance Center (NQAC) Singapore, 29 Quality Road, Singapore 618802, Singapore; wai-chinn.chan@rdsg.nestle.com (W.-C.C.); xiaoyu.wang@rd.nestle.com (X.W.)

**Keywords:** HRMS, Orbitrap, multi-residues, climate change, mycotoxins, pesticides, plant toxins

## Abstract

The consequences of climate change along with diverse food regulations and agricultural practices worldwide are complexifying the occurrence and management of chemical contaminants in food. In this context, we present an ultra-high-performance liquid chromatography high-resolution mass spectrometry (LC-HRMS) approach for the simultaneous identification and quantitation of over 1100 pesticide residues, mycotoxins, and plant toxins in cereals and fruits and vegetables. Analytical conditions were optimized to maximize the scope of the targeted molecules, the reliability of compound identification, and quantification performance within a single method. The method was further transferred and validated in another laboratory to assess its ruggedness. Validation according to the SANTE 11312/2021v2 guidelines showed that 92% and 98% of the molecules fulfill the quantification criteria at the lowest validated level in the cereals and fruits and vegetables groups, respectively. Analysis of fifteen certified reference materials led to a 96% satisfactory rate of z-scores confirming method’s competitiveness. Furthermore, the occurrence of these contaminants was studied in 205 cereals and grains samples collected worldwide. The low µg/kg quantification limits make this LC-HRMS method a valuable tool to ensure compliance toward regulations and to screen for non-regulated substances for which occurrence data are crucial for an appropriate risk evaluation.

## 1. Introduction

In 2020, the Food and Agriculture Organization (FAO) of the United Nations stated that the demand for pesticides is expected to increase due to population growth and the need for intensified crop production areas. Climate change, including higher temperatures, heat stress, and changes in rainfall patterns, is also projected to contribute to the rise of pest populations, necessitating greater pesticide use to maintain agricultural productivity [1]. In November 2023, the European Parliament rejected the European Commission’s proposal for the “Pesticides Regulation” as it would affect the viability and competitiveness of European farmers [2]. The primary objective of this regulation was to mitigate related health and environmental risks by reducing pesticide use by 50% by the year 2030. It is therefore reasonable to assume that the increased demand for food production along with climate change will lead to more occurrences of pesticide residues in food products. Meanwhile, climate change has already altered the distribution of toxigenic fungi and the appearance of mycotoxins in crops from different geographical areas in a unpredictable way [3]. Typically, the migration of the major mycotoxins into areas that lack the appropriate capacity for surveillance and risk management is of concern today. The effects on modified and emerging mycotoxins occurrence and the subsequent impacts on human health remain to be investigated as well [1]. As for pesticides detected in the same food product, the co-occurrence of several mycotoxins is observed on a regular basis [4,5]. These two classes of contaminants—pesticides and mycotoxins—are, today, the main ones regularly subjected to notifications in the European Rapid Alert System for Food and Feed (RASFF) [6]. Practices currently promoted by regenerative agriculture initiatives (less fungicides and herbicides, crop rotation, minimum or no tillage, cover crop, organic fertilizer, etc.) also impact the occurrence of contaminants in harvested crops [7]. Consumers’ exposure to contaminants is also influenced by their evolving diets. Typically, the increased consumption of plant-based products as alternatives to meat caused the WHO to raise concerns regarding human exposure to natural toxins [8]. These compounds produced by plants might indeed pose a serious health threat to both humans and livestock originating either from edible plants (e.g., aromatic plants and vegetables used as ingredients) or from the co-contamination of crops when non-edible plants and/or its seeds are co-harvested [9]. Altogether, these dynamic changes along with the global food trade, where regulations vary between countries and regions, are complexifying the occurrence and management of chemical contaminants in food. In this context, the use of analytical methods that enable the simultaneous detection of several hundreds of contaminants from different classes in several types of food matrices can provide more information to assess compliance toward existing regulations, as well as food safety in relation to chronic exposure to multiple categories of molecules.

Translating this goal to analytical science perspectives reveals that liquid chromatography-tandem mass spectrometry (LC-MS/MS)-based approaches commonly developed in recent decades are reaching their limitations, with confirmatory analysis restricted to a range of 300–400 analytes per method. Thanks to its versatility and high selectivity, the shift toward liquid chromatography high-resolution mass spectrometry (LC-HRMS) for the extensive screening of a broad range of compounds has become a key complementary option to traditional LC-MS/MS methods [10,11,12,13,14,15]. Indeed, in the full scan mode, theoretically, LC-HRMS has the potential to monitor an unlimited number of molecules simultaneously. In addition, a high resolution with accurate mass measurement capabilities can help differentiate between compounds with similar masses or isobaric interferences from co-extracted matrix components, thus reducing the chances of false positives. Non-targeted screening and retrospective analyses, both searching for the HRMS data of non-previously considered contaminants against large compounds database, have been often mentioned as added values of HRMS technology. The competitiveness of LC-HRMS versus LC-MS/MS in terms of sensitivity has significantly improved over time, as recently reported in a study focusing on pesticides, natural toxins, and veterinary drugs [16]. Additionally, these approaches have the potential to reduce costs and analysis times in routine laboratory environments focusing on compliance, provided that the extraction step(s) and analytical conditions are suitable for the analytes under survey. 

**Figure 1:**
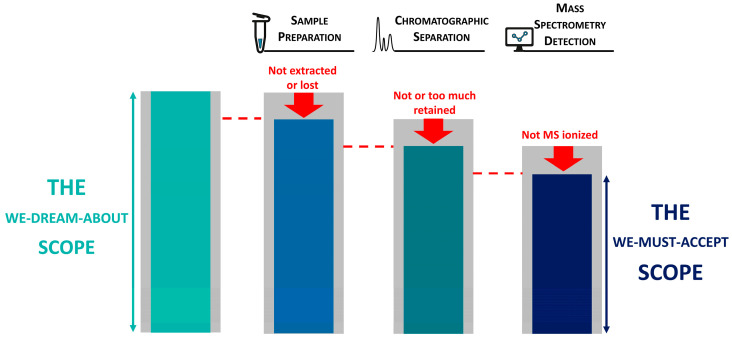
Multi-residue methods: from the “we-dream-about” scope to the “we-must-accept” scope.

However, the development of such a wide scope, multifamily method is obviously a matter of compromise. Irrespective of the mass analyzer (MS/MS or HRMS), these methods remain limited by the sample preparation, the chromatographic separation conditions, the MS ionization efficiency, and, ultimately, the physico-chemical properties of the analytes. In this context, only generic conditions allow us to extend the scope of compounds monitored to a higher level. The “we-dream-about” scope of application needs to be revisited based on observed method performances into a “we-must-accept” scope (Figure 1), and method development should focus on maximizing the number of compounds analyzable. 

In this study, we apply a QuEChERS-based [17] LC-HRMS approach for the targeted analysis of 1113 contaminants comprising 982 pesticides, 52 mycotoxins, and 79 plant toxins in cereals and grains, fruits, and vegetables (listed in Supplementary Data S1). Several inputs were considered to establish a comprehensive list of pertinent target analytes. First, natural toxins already included in a previous study were again considered [9]. They comprised mycotoxins and plant toxins (pyrrolizidine alkaloids and tropane alkaloids) for which the maximum levels (MLs) are already in place [18,19] or under discussion in the EU. Also, modified forms and emerging mycotoxins for which the occurrence data were recently reported in scientific articles or requested in the yearly EFSA calls for occurrence data [20], and other plant toxins with known toxicological effects (carcinogenic, mutagenic, etc.) were listed. For pesticides, an extensive data review focusing on EU regulations [21], private service lab analytical capabilities, EU pesticides monitoring programs in 2011–2018 [22], scientific articles [15], and recently registered pesticides [21,23] was considered. We discuss the work that was undertaken to maximize (i) the number of molecules under survey, (ii) the reliability of compound identification, and (iii) the quantification capacity, all being achieved in one single injection of food extract. The method was initially developed in an R&D laboratory located in Switzerland, prior to implementation in a routine laboratory at Singapore. Its real-life applicability is further demonstrated through a survey on 205 cereal-based samples collected worldwide.

## 2. Materials and Methods

### 2.1. Chemicals and Reagents

For sample preparation, LC gradient grade solvents (water, methanol, acetonitrile, and DMSO) were from Merck (Darmstadt, Germany). Ready-to-use QuEChERS extraction salt mixtures (magnesium sulphate (4 g), sodium chloride (1 g), trisodium citrate dihydrate (1 g), and disodium citrate sesquihydrate (0.5 g)) were supplied by Agilent (Geneva, Switzerland) while dispersive solid phase extraction salts (d-SPE, made of 150 mg of C18EC and 900 mg of magnesium sulphate) were from UCT (Bristol, PA, USA). 

For LC-HRMS analysis, Biosolve ULC/MS grade water, methanol, acetonitrile, and 2-propanol were purchased from Chemie Brunschwig AG (Basel, Switzerland). Ready-to-use ammonium formate solution (10 M in H_2_O) and LC-MS grade formic acid were supplied by Merck.

All individual analytical standards were purchased from Merck, LGC (Wesel, Germany), PhytoLab (Vestenbergsgreuth, Germany), Romer Labs (Tulln, Austria), Santa Cruz Biotechnology (Heidelberg, Germany), Angewandte Synthesechemie Adlershof (Berlin, Germany), and Oskar Tropitzsch (Marktredwitz, Germany). Smart Solutions™ v700 LC PestiMix was from LGC.

### 2.2. Stock Solutions

Individual stock standard solutions were either prepared from powdered standards at ca. 1 mg/mL in appropriate dilution solvents or available as ready-to-use solutions. For each stock standard solution prepared from powdered standard, the final concentration was calculated taking into consideration the purity, moisture, and salt of the standard according to the supplier’s certificate of analysis (CoA).

The preparation of the different working standards solutions is detailed in Supplementary Data S2. Basically, three natural toxins’ working standard solutions were prepared and stored at −20 °C for one year. They were: (a) The “plant toxin” solution that comprised 79 individual toxins at 0.1 µg/mL (56 pyrrolizidine alkaloids and 19 tropanes alkaloids) or 1 µg/mL (4 isoquinoline alkaloids) in methanol; (b) the “ergot alkaloids” solution that comprised 12 individual compounds at 0.1 µg/mL in a mixture water–acetonitrile (1 + 9, v + v); and (c) the “multi-mycotoxin” solution that comprised 40 compounds at 0.05, 0.5, or 5 µg/mL in acetonitrile. For pesticides, four in-house working solutions containing 40 to 80 individual substances were prepared at 5 µg/mL in acetonitrile and stored at −20 °C for up to one year. In addition, five ready-to-use ampoules at 5 µg/mL in various solvent mixtures were available from the ready-to-use Smart Solutions™ v700 LC PestiMix Kit from LGC. Finally, single “multi-pesticide” working standard solutions were prepared each day of analysis at 0.5 µg/mL in acetonitrile combining the 4 in-house pesticide mixes along with the five ready-to-use PestiMix ampoules. Six-level calibration solutions were also prepared each day of analysis in six separate LC-MS vials by mixing the “plant toxin”, “ergot alkaloid”, “multi-mycotoxin”, and “multi-pesticide” stock solutions, ultimately representing 1113 compounds.

### 2.3. Samples Collection

Cereal samples (grains or flour of wheat, corn, rice, oat, rye, and barley) and fruit and vegetable purées were collected from diverse internal facilities worldwide or from local supermarkets. Cereal grains were finely ground (ZM200 mill, Retsch, Haan, Germany), ensuring a maximum particle size of 1 mm. Fifteen former proficiency test materials still commercially available as quality control items were obtained from FAPAS^®^ (FERA Science Ltd., York, UK) and were pesticides in wheat flour (T09149QC, T09145QC, T09154QC), brown rice (T09148QC), barley flour (T09157QC, T09147QC), basmati rice (T09144QC), cucumber puree (T19370QC), tomato purée (T19369QC), banana puree (T19378QC), strawberry puree (T19380QC), broccoli puree (T19374QC), and dried apple (T19376QC), and mycotoxins in maize flour (T04448QC) and oat flour (T04470QC).

### 2.4. Sample Extraction

The European Norm EN 15662:2018 (QuEChERS-based procedure [17]) was used with minor modifications. Each sample (5.00 g ± 0.02 g) was weighed into a 50 mL polypropylene tube (Becton Dickinson, Le Pont de Claix, France). Water (10 mL) and a ceramic homogenizer were added, and the tubes were vigorously hand shaken for a few seconds. Acetonitrile (10 mL) was then added, and the tubes were mechanically shaken at 1500 rpm for 3 min (Spex SamplePrep GenoGrinder, Stanmore, UK). Ready-to-use QuEChERS salts were poured into each tube to initiate phase separation. The suspension was briefly hand shaken to prevent any lump formation and then placed onto a mechanical shaker at 1500 rpm for 3 min. After centrifugation (4000× *g*, 10 min, Heraeus Multifuge, Thermo Scientific, Ecublens, Switzerland), an aliquot (6 mL) of the upper acetonitrile phase was transferred into a 15 mL polypropylene tube containing the d-SPE salt mixture, and then placed onto a mechanical shaker at 1500 rpm for 3 min. After centrifugation (4000× *g*, 10 min), 1 mL of the supernatant was transferred into a new 15 mL polypropylene tube and evaporated to dryness under a stream of nitrogen at 30 °C. Residues were first reconstituted with acetonitrile (400 µL), vortexed for ca. 5 s, and further sonicated for 2 min in an ultrasonic bath (Ultrasonic cleaner, VWR, Nyon, Switzerland). Water (100 µL) was finally added, and the resulting mixture was vortexed for ca. 5 s and sonicated for 2 min in an ultrasonic bath. The extract was then transferred into a snap-lock tube and centrifuged (17,000× *g*, 10 min, RT, centrifuge Heraeus Frisco 17, Thermo Scientific) to facilitate the precipitation of remaining impurities. The clean extract was then transferred into a glass LC vial and injected in the LC-HRMS system.

### 2.5. LC-HRMS Analysis

Analyses were conducted on a Vanquish Horizon LC system coupled to a Orbitrap Exploris 480 HRMS instrument (Thermo Fisher Scientific, Bremen, Germany). The chromatographic separation was performed on a BEH C18 column (150 × 2.1 mm, 1.7 μm) (Waters, Milford, MA, USA) heated at 50 °C using a 400 μL/min flow rate. An autosampler program was used for injection to improve the peak shapes of early eluting compounds. Thus, 15 µL of water was added to the 1 µL of sample extract and the resulting mixture was mixed into the syringe before being injected. Acidic mobile phases were water (mobile phase A) and methanol (mobile phase B) both containing 0.5 mM of ammonium formate and 0.1% formic acid. A 22 min gradient was set as follows: 0–1.0 min (5% B); 1.0–18.0 min (up to 100% B); 18.0–19.5 min (100% B); 19.5–19.6 min (down to 5% B); and 19.6–22.0 min (5% B). 

The acquisition was performed in the full scan MS/data-dependent MS2 (FS-ddMS2) mode. The Orbitrap Exploris 480 instrument was equipped with a heated-electrospray ionization probe operating in positive or negative modes (two separate injections). Source and MS parameters were as follows: sheath gas and auxiliary gas flow rate of 50 and 10 arbitrary units, respectively; sweep gas flow rate of 1 arbitrary unit; spray voltage of 3.5 kV for the positive mode and 2.5 kV for the negative mode; ion transfer tube temperature of 250 °C; and vaporizer temperature of 350 °C. The normalized automatic gain control (AGC) target value was set to 300% and maximum injection time (IT) at 50 ms, with a resolving power of 120,000 FWHM (at *m/z* 200) in the full-scan MS mode at the ranges of 98–920 Da and 95–700 Da in the positive and negative modes, respectively. Data-dependent fragmentation (dd-MS2) was set with a resolving power of 15,000 FWHM (at *m*/*z* = 200), isolation window of 1.0 Da, normalized AGC at 100%, maximum IT of 50 ms, dynamic exclusion duration of 3 s, and intensity threshold of 1 × 10^5^ cps. An inclusion list of selected parent ions with their respective retention times (RT window: 1 min) was used with optimized normalized collision energy (NCE) to specify the priority compounds for MS2 experiments.

### 2.6. HRMS Mass Spectral Library

Each individual standard solution was first injected without any chromatography column (flow injection) at concentration levels in the 0.1–1 µg/mL range. Product ion spectra were collected in both ionization modes at 10 different NCEs (from 10 to 100) for each precursor of interest (e.g., protonated and ammonium adducts in the positive ionization mode; deprotonated and formate adducts in the negative mode). When no adducts were detected, in-source-fragments were investigated. All spectra were eventually populated in mzVault^TM^ 2.3 software (Thermo Fisher Scientific) and were automatically recalibrated based on the precursor ion exact mass. 

### 2.7. Data Acquisition and Data Treatment

A compound database was created from the mzVault^TM^ library as follows. For each analyte, an optimal NCE was determined, for which the intensity of the precursor was less than ca. 20% of the most abundant fragment intensity [9]. The five most intense fragments, excluding the parent one, at the chosen NCE were selected. This compound database was further incorporated in a quantitative TraceFinder^TM^ 5.1 software (Thermo Fisher Scientific) workflow and considered for analytes identification (Supplementary Data S3).

Data acquisition and data processing were carried out using TraceFinder^TM^ 5.1 software. To facilitate their interpretation, all the data were further exported in a csv format and reorganized in Microsoft Excel (for Microsoft 365 MSO (Version 2407)) using a dedicated internally developed IT tool based on R software (R-4.3.2 version) [24].

### 2.8. Identification Criteria

Identification criteria were adapted from the SANTE 11312/2021v2 [25] document and were: (i) a signal observed for the precursor ion (protonated molecule, deprotonated molecule, adduct ion, and ion source fragment) of the related compound within a mass extraction window of 5 ppm and with a retention time corresponding to that of the analyte in solvent with a tolerance of ±0.1 min, and (ii) a minimum of two fragments with a mass accuracy below 5 ppm. For hardly fragmentable compounds, only one product ion was requested. To provide additional support for identification, the relative intensities between fragments obtained in the matrix to those obtained for calibration solutions or a positive QC sample initially fortified with reference analytical standard solutions was considered. 

### 2.9. Quantification

For each compound positively identified in a sample, quantification was performed by means of an external calibration. The calibration curve was drawn for each individual compound by plotting the peak area of the analyte (= y axis) against the concentration of the analyte (= x axis, in µg/kg equivalent in-sample concentration) using calibration solutions ranging from Cal 0 to Cal 5 (corresponding levels provided in Supplementary Data S4). A 1/x^2^ weighing factor was applied. The linearity was considered as satisfactory when: (a) the determination coefficient r^2^ was higher than 0.98 and (b) the deviations of the back-calculated concentrations of the calibrants from the true concentrations, using the calibration curve, were not more than ±20% [25]. The mass fraction, w, of a positive compound present in the sample was expressed in µg/kg using the following equation:

$$w=\frac{A_{\mathrm{analyte}}-I}{S}$$

where w is the mass fraction of a given analyte in the sample (in μg/kg), *A* is the MS peak area of the given analyte, and *I* and *S* represent the intercept and slope of the 1/x^2^ weighing factor regression line, respectively.

### 2.10. Method Performances

Two laboratories equipped with the same instruments were involved in this study. Laboratory 1 (Switzerland) developed the method on the full set of compounds, whilst Laboratory 2 (Singapore) performed a method validation using the developed protocol without any modifications to efficiently assess method ruggedness. The commodity groups “high starch and/or protein content and low water and fat content” and “high water content” were validated according to the SANTE 11312/2021v2 guidelines [25]. First, an initial full validation was conducted on at least one representative sample from both commodity groups (wheat flour, corn flour, and fruit purees) for which test portions were fortified with all analytes at two different levels (Level 1 and Level 2) performing six replicates in a single day. Repeatability (CV_r_) and recovery (Rec) were derived from the experiments. Then, an on-going validation was performed by including in each batch of routine analysis at least one sample from these commodity groups fortified in duplicate at Level 1. This led to 44 individual results (*n* = 22 samples of wheat, corn, oat, and rice flour) and 12 individual results (*n* = 6 samples of fruit purees) for each analyte for each of the two categories studied. Repeatability, intermediate reproducibility (CV_iR_), recovery, and measurement uncertainties (U) were then calculated according to ISO 5725–2 [26]. Recovery values at the fortified concentrations were calculated from the median values of spiked experiments performed under intermediate reproducibility (iR) conditions. Non-fortified samples were systematically analyzed to check for any potential occurrence. For naturally contaminated samples, the native concentration(s) obtained from unspiked samples was/were subtracted from the spiked value(s) obtained. The overall uncertainty was obtained by combining precision and recovery contributions [27]:

$$U\left(\%\right)=2\times \sqrt{RSD{\left(iR\right)}^{2}+RSD{\left(Rec\right)}^{2}}$$

where *U* (%) is the expanded uncertainty at the 95% confidence interval; *RSD*(*iR*) is the relative standard deviation of intermediate reproducibility and *RSD*(*Rec*) is the relative standard deviation of recovery. The LOQ for each analyte was defined as the lowest successfully validated level [25]. To obtain insights into the validity of the method, 15 certified reference materials were also analyzed in a single replicate.

## 3. Results

### 3.1. Maximizing the “We-Must-Accept” Scope of Compounds

#### 3.1.1. LC Separation and Sample Extraction 

A 22 min LC run using acidic mobile phases on a 15 cm reversed-phase (RP) C18 column was considered as a good compromise to retain a broad range of chemicals with various polarities, but also to separate a maximum of isomers and isobaric compounds by using a smooth gradient slope whilst keeping an acceptable run time. Still, such a “long” run was expected to minimize potential matrix effects generated from co-extracted matrix components. Using these conditions, compounds were eluted at retention times (RTs) between 0.9 min and 18.6 min with peak widths around 0.1 min (6 s) for most of the compounds, which was sufficient to obtain 10–12 data point per peak.

**Figure 2:**
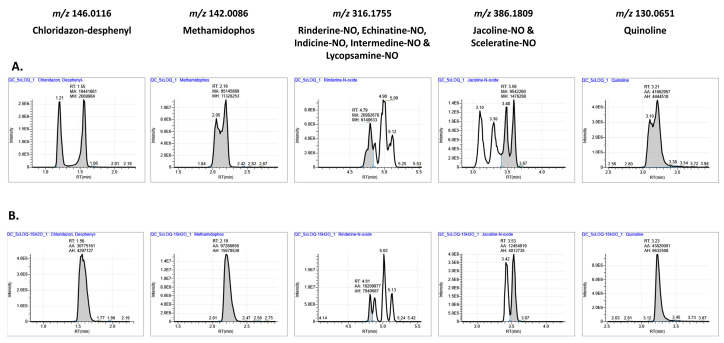
Extracted ion chromatograms (±5 ppm) of several ions. Standard solution (injection volume: 1 µL, 5–25 ng/mL in acetonitrile-water (8 + 2, v + v)) directly injected (**A**) or using an autosampler program (addition of 15 µL water in the injection syringe) (**B**).

These classical LC conditions were combined with a generic extraction procedure based on the QuEChERS procedure followed by a dispersive SPE-clean up using MgSO_4_ and endcapped C18 salts. The addition of other cleanup salts, such as PSA, led to an unacceptable loss of compounds. After evaporation (concentration factor ×2), final extracts needed reconstitution with a high amount of organic solvent to ensure the solubility of the most apolar molecules, such as enniatins and avermectins. Consequently, the injection solvent was composed of 80% acetonitrile, while the initial mobile phase condition was 95% water. This obviously led to a poor peak shape of early eluting compounds, such as methamidophos, chloridazon-desphenyl, quinoline, or pymetrozine, even when using a low injection volume (1 µL) (Figure 2A). To circumvent such an issue, an autosampler injector program was set up: for each injection, 15 µL of water were added to 1 µL of sample extract, and the resulting mixture composed of 95% water was mixed into the syringe before being injected. The effect on peak shape became immediately evident, as depicted in Figure 2B, particularly for the numerous co-eluting isomers of pyrrolizidine alkaloids, ultimately leading to easier identification. The use of such an autosampler program avoids an additional aqueous dilution step that would compromise the overall method sensitivity. In total, around 5% of the total scope of compounds (ca. 50 compounds) eluting before 5 min was positively impacted by this autosampler program. 

Using these conditions, the “we-must-accept” scope was composed of 1113 compounds classified as 982 pesticides, 79 plant toxins, and 52 mycotoxins. Moreover, 50 compounds initially present in the Smart Solutions™ v700 LC PestiMix Kit from LGC were removed from the scope of this study and were mostly GC amenable compounds or requested dedicated extraction conditions (Supplementary Data S2).

#### 3.1.2. HRMS Acquisition

The acquisition mode was similar to the one described in our previous studies limited to plant toxins analysis [9] or veterinary drugs residues [28]. A full-scan mode (over the *m*/*z* 98–920 range in the positive mode and *m*/*z* 95–900 in the negative mode) at a resolution (Rs) of 120,000 FWHM (at *m*/*z* 200) was selected for improved selectivity. A data-dependent product ion scan (ddMS2, Rs 15,000) was then triggered on the five most intense ions (top-five experiments). To favor the fragmentation of targeted analytes over other ions, an inclusion list containing analyte monoisotopic masses for the parent ion and/or most abundant adducts as well as their retention times (RTs) and their optimal collision energies both for the positive and negative ionization modes was key.

**Figure 3:**
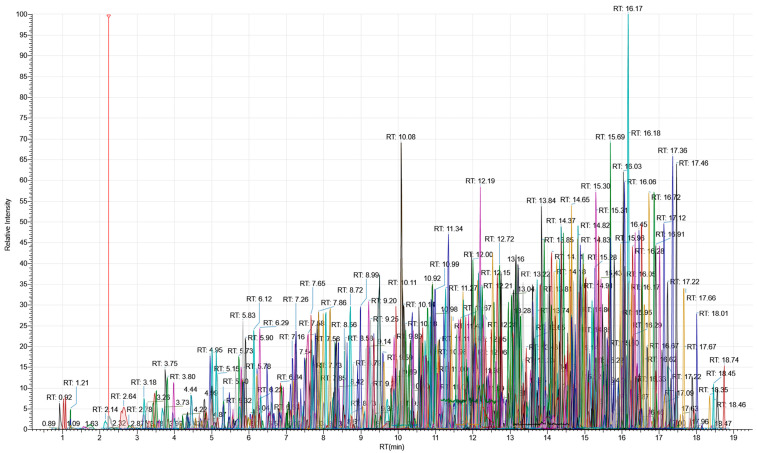
Extracted ion chromatograms (±5 ppm) in the positive ionization mode for 1032 compounds prepared at 1–100 ng/mL in acetonitrile-water (8 + 2, v + v)) (corresponding to the lowest calibration point).

Instrument software default acquisition parameters were optimized to improve the sensitivity of compounds at trace levels [28]. Typically, the total number of ions that enter Orbitrap was maintained by the AGC from the C-Trap along with the IT. For the full scan experiment, AGC was finally set at 3 × 10^6^ (normalized AGC = 300%; 100% by default) with a maximum IT time of 50 ms (100 ms by default). For ddMS2 experiments, constant parameters (Rs: 15,000; IT time: 50 ms) were perceived as a good compromise between the quality of the spectra generated and the overall cycle time. Using such conditions, a theoretical full-scan MS mode at Rs = 120,000 followed by 5 ddMS2 led to a ca. 500 ms cycle time, ensuring, theoretically, at least 10 data points per peak (Figure 3).

### 3.2. Maximizing Compound Identification Reliability

#### 3.2.1. In-House Accurate Mass Spectral Library

**Figure 4:**
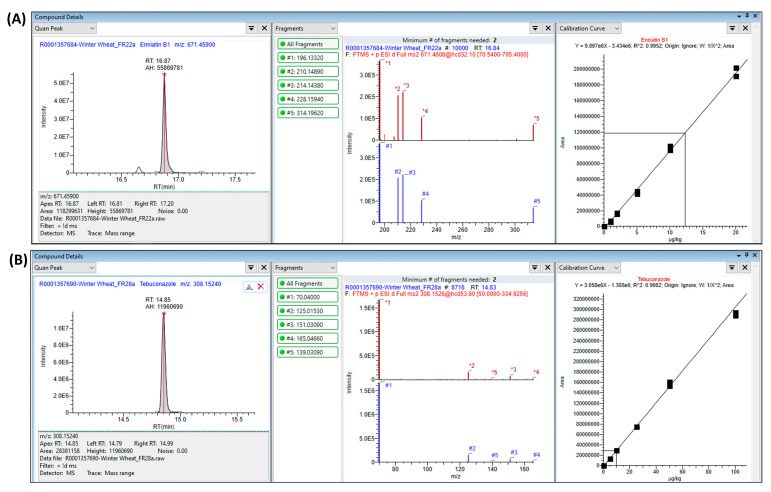
Identification and quantification of Enniatin B1 (**A**, 12.3 µg/kg) and Tebuconazole (**B**, 9.7 µg/kg) in two winter wheat samples. Extracted ion chromatograms from precursor ions (*m*/*z* 671.4590 and 308.1524), fragmentation spectra (#1–#5 masses refer to the theoretical ones; *1–*5 masses refer to the experimental ones), and a six-point calibration curve.

The foundation of our study relies on a high-quality and accurate in-house mass spectral library with the retention time created using pure reference analytical standards. HRMS libraries are indeed pivotal for the unambiguous identification of molecules during a targeted analysis. The acquisition mode used in this study allowed for a direct identification based on the high-quality fragmentation spectrum that was compared with the five most intense fragments selected from our mass spectral library. As shown in Figure 4A, an intense signal in the full scan mode at 16.87 min (*m*/*z* 671.4608) is attributed to Enniatin B1 (*m*/*z* 671.4590, mass accuracy: 2.6 ppm) thanks to a high-quality fragmentation spectrum collected at RT 16.84 min (NCE of 32.1). The five fragments detected matched perfectly with the five Enniatin B1 fragments registered in our in-house HRMS mass spectral library (mass accuracy: 0.27–0.96 ppm). Similarly, Tebuconazole was unambiguously identified (RT: 14.85 min, *m*/*z* 308.1526, and mass accuracy: 0.6 ppm) in another wheat sample (Figure 4B). In both cases, 15 identification points were obtained (precursor ion at ±5 ppm for 1.5 pts, 5 product ions at ±5 ppm for 5 × 2.5 pts, and retention time tolerance of ±0.1 min for 1 pt), largely exceeding the minimum of five identification points required by EC 2021/808 [29]. 

#### 3.2.2. Identification of Isomers and Isobaric Ions

The simultaneous identification of hundreds of substances requires the capability to differentiate isomer ions having the same elemental composition and *m*/*z* and isobaric ions having the same nominal mass but a different elemental composition. Sufficient mass resolving power and/or chromatographic resolution are usually required to unequivocally identify them. In this study, 79 pairs or groups of isomers were identified leading to different scenarios. First, 60 pairs/groups were well separated by their respective retention times (RTs), allowing an unambiguous identification and further quantification. Among those, five pairs were not fully chromatographically resolved (double peak, ΔRT ~0.05–0.1 min), but the software algorithm still allowed an appropriate integration. Secondly, five pairs of isomers could not be separated (full-scan MS mode) with ΔRT <0.05 min (3 s), but exhibited different MS2 fragmentation spectra, leading to an unambiguous identification (Pebulate and Vernolate, Propazine-2-hydroxy and Terbutylazine-2-hydroxy, Desmetryn and Simetryn, Prometon and Secbumeton, and Atropine-N-oxide and Convolamine). In the case of positive findings, the subsequent quantification using the corresponding individual reference standard solution had to be considered. Finally, 14 pairs of isomers could not be distinguished by either the retention time or the fragmentation pattern. Therefore, the compounds were analyzed as the “sum of” (e.g., Indicine and Lycopsamine, Allethrin and Bioallethrin, or Fenoxaprop-ethyl and Fenoxaprop-P-ethyl) considering the equivalent MS response factor. 

The least absolute mass difference (expressed in ppm) of two masses that Orbitrap can resolve at a 50% peak valley using a resolution of 120,000 FWMH (at *m*/*z* 200) was calculated [30]. Under our conditions, theoretical calculations indicated that two ions could be resolved when the mass difference was at a minimum of 12 ppm at *m*/*z* 200 and up to 25 ppm at *m*/*z* 900. As expected, the resolution decreased as the measured mass increased. Among the 76 pairs of isobaric ions with a theoretical delta <25 ppm mass measurement, only three pairs co-eluted (ΔRT < 0.1 min), hampering a direct quantification using a global standard mixture. They were: (a) Flutianil (C_19_H_14_F_4_N_2_OS_2_, *m*/*z* 427.0556, 14.85 min) and Ipfencarbazone (C_18_H_14_Cl_2_F_2_N_4_O_2_, *m*/*z* 427.0535, 14.85 min) with Δ *m*/*z* = 4.9 ppm; (b) Fenfuram (C_12_H_11_NO_2_, *m*/*z* 202.0863, 10.98 min) and Terbuthylazine-desethyl (C_7_H_12_ClN_5_, *m*/*z* 202.0850, 10.94 min) with Δ *m*/*z* 6.2 ppm; and (c) Pyraoxystrobin (C_22_H_21_ClN_2_O_4_, *m*/*z* 413.1263, 15.22 min) and Cyflufenamid (C_20_H_17_F_5_N_2_O_2_, *m*/*z* 413.1283, 15.25 min) with Δ *m*/*z* 4.8 ppm. More challenging was to identify the possibility to have ion(s), where their minor isotopes (e.g., ^37^Cl) or other adducts (e.g., [M + NH_4_]^+^) matched other selected precursor ion(s). Particularly, fenoxanil was monitored through its protonated precursor ion ([M + H]^+^ *m*/*z* 329.0818 for 14.48 min), but its ammonium adduct ([M + NH_4_]^+^; *m*/*z* 346.1084) was significant as well, and its mass was close to the Nitralin ion (*m*/*z* 346.1067, 14.46 min), with a delta mass measurement of 4.7 ppm and the same retention time.

**Figure 5:**
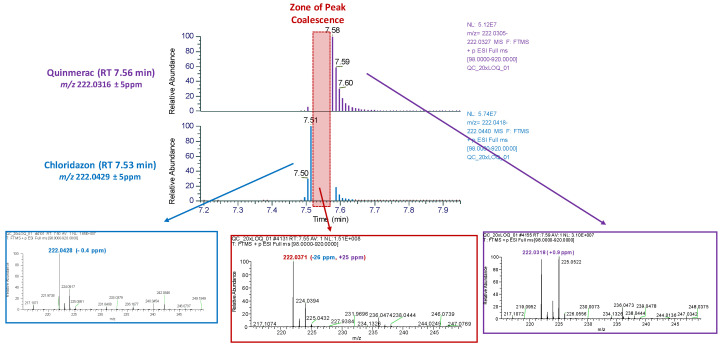
While Quinmerac (C_11_H_8_ClNO_2_, *m*/*z* 222.0316, and RT = 7.56 min) and Chloridazon (C_10_H_8_ClN_3_O, *m*/*z* 222.0429, and RT = 7.53 min) have a delta mass measurement of 51 ppm, the co-elution region presents a mass shift for both compounds. In the peak coalescence zone (in red), there is a mass measurement error of +25 ppm for Quinmerac and −26 ppm for Chloridazon.

Peak coalescence [31] was also observed for a few co-eluting isobaric compounds, despite a value of Δ *m*/*z* > 25 ppm. During the time interval, where two ions are introduced simultaneously into the ion trap, the near coincidence of their secular frequencies results in an ion cloud overlap and in the distortion of the measured frequency, ultimately producing a mass measurement error. In our study, we observed an unexpected larger mass error for a few co-eluting isobaric compounds, despite Δ *m*/*z* > 25 ppm. While Quinmerac (C_11_H_8_ClNO_2_, *m*/*z* 222.0316, and RT = 7.56 min) and Chloridazon (C_10_H_8_ClN_3_O, *m*/*z* 222.0429, and RT = 7.53 min) had a delta mass measurement of 51 ppm, the co-elution region resulted in a mass shift for both compounds. In the peak coalescence zone (Figure 5), a mass measurement error of +25 ppm for Quinmerac and −26 ppm for Chloridazon was observed, far above the 5 ppm mass extraction windows. Similarly, Isazofos (C_9_H_17_ClN_3_O_3_PS, *m*/*z* 314.0490, and RT = 13.79 min) and Triazophos (C_12_H_16_N_3_O_3_PS, *m*/*z* 317.0723, and RT = 13.82 min) exhibited a delta mass measurement of 74 ppm, but the co-elution region resulted in a mass shift of +9 ppm and −9 ppm for both compounds, respectively. 

#### 3.2.3. Anticipate Needs for Scope Extension 

The acquisition mode was built to maximize the chance to continuously extend the scope of molecules targeted as well as collecting the required information for suspect screening or retrospective data analysis. First, for each targeted compound in the inclusion list, a 1 min time window around its retention time was allocated to favor mass fragmentation over other ions. Second, intense ions that were not included in the targeted compound list still had the opportunity to be fragmented, this non-targeted fragmentation being performed at three different collision energies (NCE 20-40-60) further combined in a single MS2 spectra. As example, the acquisition of a wheat extract injected under the described conditions in the positive ESI mode led to 9720 MS2 spectra either from the precursor ions mentioned in the inclusion list (*n* = 152 ions) or from ions not initially targeted (*n* = 1116 ions). This opens avenues for the addition of new compounds in the scope of our study, without compromising identification as well as for suspect screening or retrospective data analysis.

### 3.3. Maximizing the Quantification Capability 

Pros and cons of quantification approaches, such as isotopic dilution, matrix-matched calibration, procedural calibration, standard addition, or external calibration applied in multi-residue methods, have been discussed in the literature already [16,32,33]. In this study, the compromise between method performance, cost of analytical standards, and sample throughput for efficient turn-around times in high routine environments was found adequate when using external calibration curves prepared on a daily basis. Indeed, as demonstrated by the method performance described later, matrix effects were well limited by the long analytical run used (22 min), the low amount of sample extract injected in the MS, and the natural high volume of water typically contained in some products, like fresh fruits and vegetables.

**Figure 6:**
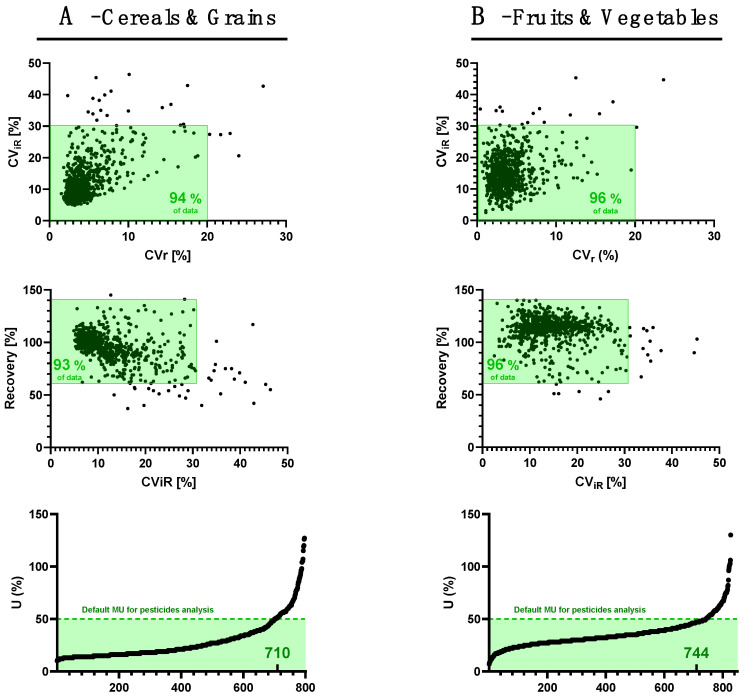
Repeatability, intermediate reproducibility, recovery, and measurement uncertainties at the lowest fortification levels (e.g., pesticides at 0.005 mg/kg, aflatoxins, and plant toxins at 1 µg/kg) in the two commodity groups: (**A**): cereals and grains; (**B**): fruits and vegetables.

The method was further deployed in Laboratory 2, and a full validation study was conducted on 860 molecules over the 1113 analytes initially scoped. The validation focused on 730 pesticides, 51 mycotoxins, and 79 plant toxins, ultimately corresponding to 841 targets considering the 19 molecules being reported as “sum of”. The on-going validation was performed at a single fortification level (targeted LOQ) gathering more than 40,000 individual measurements that were evaluated based on the following criteria: (a) false negative rate (<5%); (b) CVr < 20%; (c) CV_iR_ < 30% to afford the sample variability across the study; and (d) Rec 60–140%. The LOQ was derived from these fortification experiments when all criteria were fulfilled. For the sake of clarity, the performance criteria are summarized in Figure 6, but all the data are available in Supplementary Data S5. 

Altogether, the data collected for the “high starch and/or protein content and low water and fat content" group (cereals and grains) show that 776 analytes (92%) fulfil the quantification criteria at the lowest validated level without any further action; 24 analytes (3%) fulfil the quantification criteria at a higher LOQ (referring to the initial full validation data); 34 analytes (4%) are perfectly identified at the targeted LOQ highlighting good screening capability, but fail the quantification criteria; and seven analytes (1%) had to be removed from the initial scope due to a high risk of false negatives at the targeted levels. Typically, disulfton suffered from intense in-source fragmentation leading to an *m*/*z* 89.0419 ion outside of the mass range (*m*/*z* 98–920). Not surprisingly, the performance data for patulin or tenuazonic acid were poor since the LC analytical conditions were not optimal for their detection. Finally, measurement uncertainties were below the default of 50% U [25] for 85% of the scope of compounds. In more detail, Table 1 shows that LOQs are below the lowest EU MRLs for most of the compounds, thus demystifying discussions around HRMS’s lack of sensitivity [16]. For each aflatoxin fortified at 1 µg/kg, CVr, CV_iR_, and Rec were in the ranges of 2–4%, 6–14%, and 89–94%, respectively. Similarly, the same performance parameters were in the ranges of 2–6%, 8–23%, and 73–96% for the regulated tropane alkaloids, namely atropine and scopolamine and for each of the twelve ergot alkaloids fortified at 1 µg/kg. The family pyrrolizidine alkaloids comprise 54 individual compounds, among which 35 are regulated as a sum [18]. CVr, CV_iR_, and Rec derived from fortification experiments at 1 µg/kg and in the ranges of 2–10% (median: 4%) 1–25 (median: 14.6% and one outlier at 45%), and 54–96% (median: 78%). Lower recovery data for a few molecules were explained by their polarity and the absence of a correction of losses during sample extraction. However, considering that only 35 out of the 54 PAs monitored are regulated under their sum, the method was considered as acceptable for this class of plant toxins. 

The same conclusions mostly applied for the “high-water-content” (fruits and vegetables) commodity, where 823 analytes (98%) fulfilled the quantification criteria at the lowest validated level without any further action, eight molecules (1%) fulfilled the quantification criteria at a higher LOQ, four molecules (0.5%) needed readjustments for appropriate accuracy (work in progress), and six molecules (0.7%) had to be removed from the initial scope due to a high risk of false negatives at the targeted levels. Finally, U values were below 50% for 89% of the scope of compounds.

**Figure 7:**
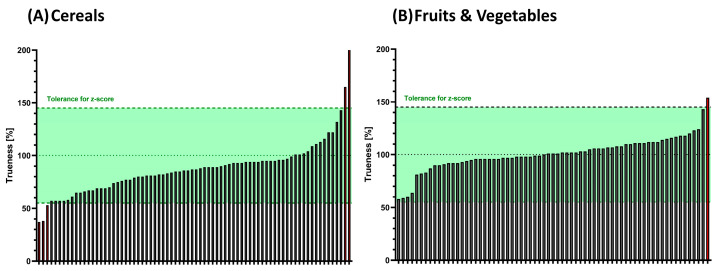
Direct quantification of contaminants in certified reference materials of (**A**) cereals and grains (*n* = 9, 78 pesticides and mycotoxins, and 2–873 µg/kg); (**B**) fruits and vegetables (*n* = 6, 69 pesticides, and 19–201 µg/kg).

A set of commercial, certified reference materials (*n* = 15) was analyzed with the LC-HRMS method (one single replicate per sample) by Laboratory 2. No false negative results were obtained since all molecules within the scope were appropriately identified in each of the 15 samples (no false negatives), according to the SANTE 11312/2021v2 criteria [25]. From a quantitative perspective (Figure 7), 141 out of 147 results (96%) exhibited z-scores within the tolerance range (−2 to +2). Out of the six results out of the tolerance range (4%), teflubenzuron in broccoli puree (z-score: 2.4), cyazofamid in barley (z-score: 2.9), and aflatoxin B1 in maize (z-score: −2.8) exceeded the tolerance values, while the performance data for these compounds were fit for purpose during the validation. The poor performance of pencycuron could finally be attributed to an issue in the reference analytical standard mixture used for quantification. Chlorpyrifos-methyl and fenazaquin in wheat were both underestimated during the assessment.

### 3.4. Mapping Occurrence of Contaminants in 205 Cereal Flours and Grains

**Figure 8:**
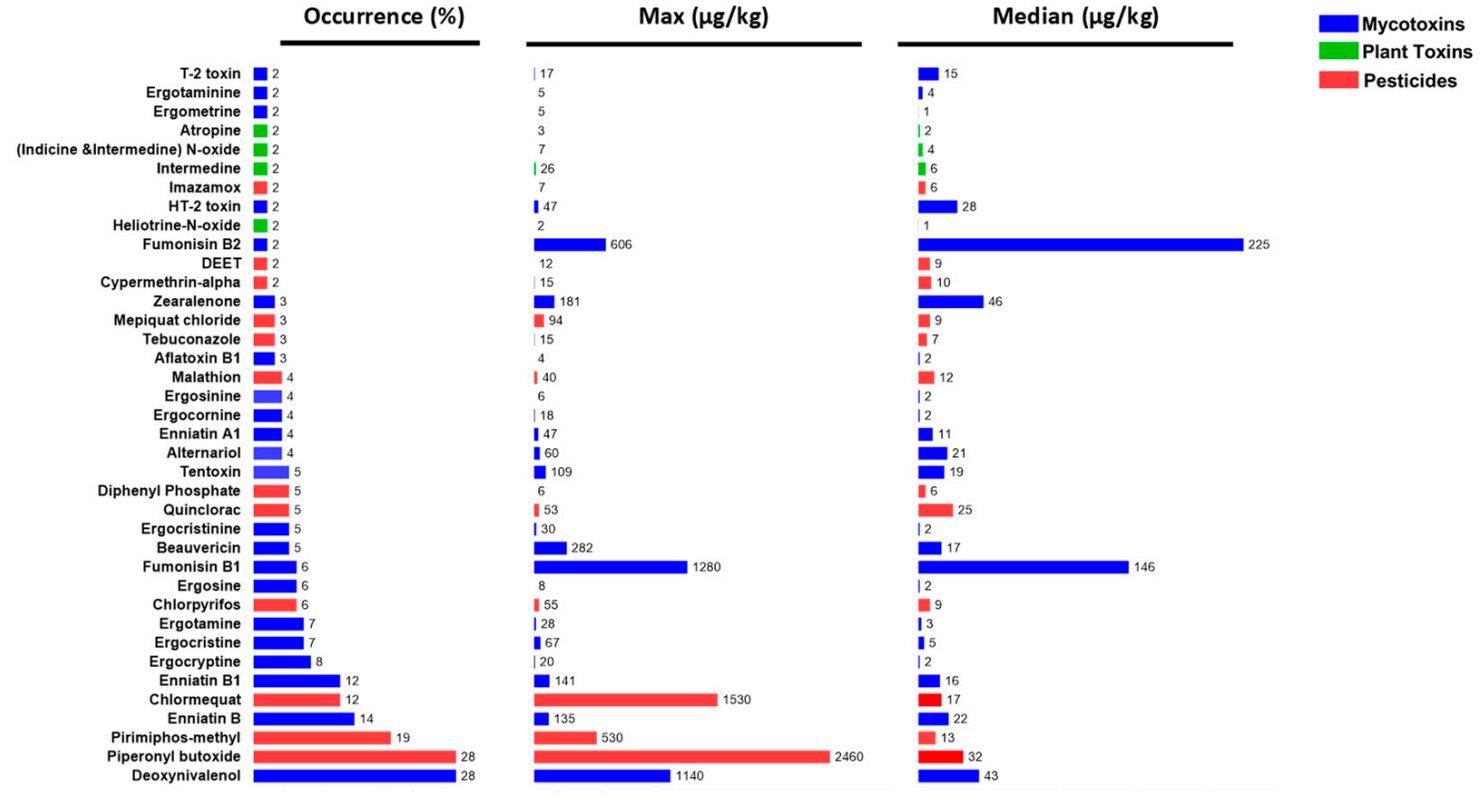
Occurrence, maximum, and median concentration levels of pesticides, mycotoxins, and natural toxins in 205 cereal-based samples (wheat, corn, oat, rice, and barley).

**Figure 9:**
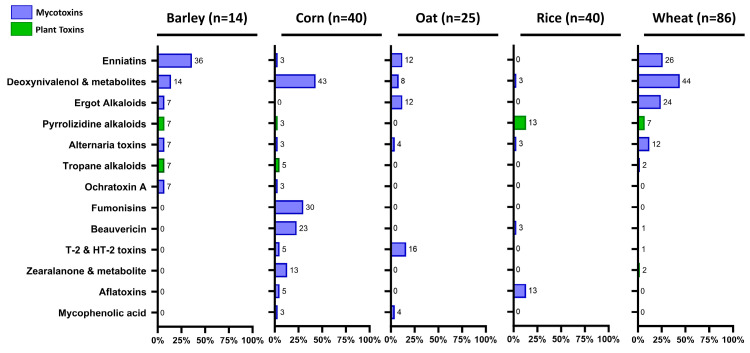
Occurrence per class of toxins (mycotoxins and plant toxins) and per commodity (*n* = 205 samples).

A set of 205 cereal flours or grain samples (wheat, corn, barley, rice, and oat) was analyzed in Laboratory 2 using the described methodology. In total, 570 out of 170’765 individual data (0.3%) were reported above the LOQ. These findings correspond to 38 pesticide residues, 33 mycotoxins, and 11 plant toxins detected in these samples. To generate such an amount of data, we roughly estimate that 5–8 different LC-MS/MS analytical methods would normally be required in a classical routine laboratory environment. The low occurrence rate, but the broad diversity of contaminants, detected was additional proof of the relevance of such a multifamily, generic, targeted workflow. To gain a deeper understanding, Figure 8 displays the occurrence, maximum, and median concentration levels of compounds reported in at lowest four out 205 samples (occurrence ≥2%). Not surprisingly, mycotoxins were the most frequently detected family of compounds. The insecticides pirimiphos-methyl and chlorpyriphos and the plant growth regulator chlormequat were the most reported pesticides. Piperonyl butoxide has no pesticide activity of its own, but it enhances the potency of certain pesticides (synergist effect), such as carbamates, pyrethrins, pyrethroids, and rotenone, and is therefore used as an adjuvant in pesticide formulations [34]. Traces of atropine and some pyrrolizidine alkaloids were sporadically detected in this set of samples as well. Occurrences of these toxins can be further split across the commodity (Figure 9), highlighting the high occurrence of enniatins in wheat and barley and beauvericin in maize compared to other molecules. Emerging mycotoxins are more often detected in agricultural commodities, but their toxicity is still under evaluation, and therefore no regulations apply yet. Typically, beauvericin, enniatins, but also sterigmatocystin, moniliformin, or diacetoxyscirpenol are listed in the last “EFSA annual call for continuous collection of chemical contaminant occurrence data in food” [20].

## 4. Discussion

The deployment of wide-scope multifamily LC-HRMS-based methods requires significant efforts, adaptations, and compromises focusing on three major ideas: generic analytical conditions, compound identification reliability, and quantification capabilities.

First, only generic conditions ranging from sample extraction to data acquisition modes allow us to maximize the scope of compounds monitored, i.e., reducing the gap between the “we-dream-about” and the “we-must-accept” scopes of application (Figure 1). For this, we made the choice to use the QuEChERS extraction method combined with a 22 min LC run using acidic mobile phases on a 15 cm RP C18 column. Final extracts were reconstituted in a high amount of organic solvent to ensure the solubility of the most apolar molecules, such as enniatins or avermectins. To circumvent the peak shape issue of early eluting compounds, we included an autosampler program to match the injection solvent composition to the initial gradient one while keeping the same quantity of extract injected in the system. All these parameters, along with the acquisition mode selected in this study, were the key to maximize the inclusion of a broad range of molecules having different physicochemical properties. However, dedicated methods will remain important for specific compounds or families of compounds that cannot be included in the “we-must-accept" scope. Some molecules cannot be retained on the RP C18 column using acidic mobile phases, and an orthogonal alkaline run for tenuazonic acid or alkaloids, such quinolizidine, low-molecular-weight tropane, or glycoalkaloids, would be more appropriate. Methods requiring hydrolysis or esterification to comply with pesticides’ regulatory requirements are always needed. The QuPPe method (Quick Method for the Analysis of Highly Polar Pesticides) also remains of interest to address very polar and non-QuEChERS-amenable pesticides in foods [35].

Second, the outstanding screening performance capabilities achieved within a single injection is due to the in-house mass spectral library, with retention times determined from pure reference analytical standards. HRMS libraries are indeed pivotal for the unambiguous identification of molecules during targeted analysis, but as well to assist with identification in suspect screening approaches. Building our own HRMS mass spectral library offers several advantages: (i) high quality and reliability of the reference spectra acquired using a standardized methodology; (ii) improve operators’ understanding of the ionization and fragmentation behavior of compounds; (iii) allow for the extended coverage of contaminants or metabolites not always included in the most up-to-date or comprehensive collections of spectra available online—for instance, only 40% of the pesticides included in our library are available from the mzCloud^TM^ database; and (iv) ensure the alignment between HRMS instruments used for mass spectra acquisition (Laboratory 1) and the ones deployed across laboratories for routine analysis and investigations (Laboratory 2). The availability of reference standards to identify and discriminate isomers and isobaric ions is key to ensure appropriate compound identification and to reduce the risk of false positive identifications. 

**Figure 10:**
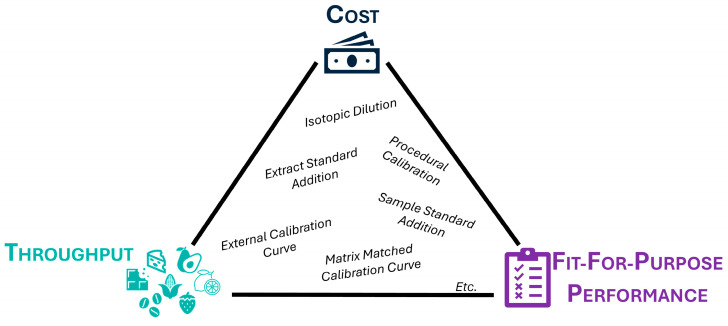
The challenge of quantification for MS-multi-residue methods: irrespective of the detector (MS/MS or HRMS), the quantification strategy is always a compromise between cost/throughput/data accuracy.

Third, the challenges created by quantification for multi-residue methods are still extensively discussed in the literature. Ultimately, irrespective of the mass analyzer (MS/MS or HRMS), the final choice remains a compromise between the overall method performance, cost of analytical standards, and sample throughput in the lab (Figure 10). In this study, the use of an external calibration curve was perceived as the best compromise, as evidenced by the overall method performance characteristics. Quantification in multi-residue analysis is never achieved for the full scope of compounds monitored [36,37,38] and new ideas are needed to deal with the minority of “out-of-norm” compounds. As pointed out elsewhere [37], such a large analyte-scope method are not intended to compete with traditional scope-restricted dedicated methods usually recognized as official reference methods or used for the measurements of contaminants on the highest metrological level (e.g., inter-laboratory certification studies of reference materials). Nevertheless, we strongly believe that the described quantitative method performances extend far beyond the typical expectations of screening methods that are commonly described in LC-MS/MS- or LC-HRMS-based multi-analyte methods in the literature. This is also highlighted by the 96% satisfactory rate of z-scores when analyzing certified reference materials, which demonstrates the competitiveness of our method compared to that employed by other proficiency test participants. It is worth noting that these participants often make use of methods specifically designed for a single type of contaminant, extensively validated, and potentially incorporating stable isotopically labeled standards for accurate quantification. 

The large amount of information available for data processing should not be neglected. Unfortunately, supplier software generally misses critical calculation features and adapted reporting tools, ultimately impairing the efficiency of standard food control laboratories that face a short turn-around time. This prompted us to develop our own automatic coded R-script capable of translating a raw export file into a comprehensive .xlsx report file in a few seconds. This in-house tool supports any screening or quantitative strategies and was thought to be user-friendly, fast, scalable, self-controlled, and flexible. Ultimately, it guides operators for fast decision making, saving up to two working days per batch of analysis [24]. Moreover, validating more than 1000 molecules in various food matrices, in accordance with the current official guidelines, is technically feasible, but comes with challenges, such as extensive data collection, significant time investment, and resource requirements, as pointed out by Steiner et al. [16]. These factors can hinder the rapid implementation of LC-HRMS in accredited laboratories. 

## 5. Conclusions

The developed LC-HRMS methodology is a highly effective filtering tool able to screen a broad range of chemical contaminants in a single analysis, to quickly focus on positive findings that are unambiguously identified according to the official criteria in a single analysis, and to immediately report contamination levels with high accuracy in most cases. It leverages the capabilities of analytics for the efficient surveillance and risk management of chemical contaminants across the supply chain, from sourcing materials to finished product manufacturing, packaging, and distribution. The low limits of quantification enable its usage as a quality control tool for regulated or soon-to-be-regulated substances to assess compliance toward regulations. In parallel, the collection of occurrence data will enable us to evaluate overall human exposure to substances, for which the risk is not fully characterized yet. 

## Figures and Tables

**Table 1:** Limits of quantification for 860 compounds in cereals and grains. LOQ is defined as the lowest fortification level for which the method performances fulfil the validation criteria. Maximum levels for infants and young children are not considered in this table.

	Compounds	*n*=	LOQ (µg/kg)	Lowest EU MRL (µg/kg) Cereals by Default If Applicable	Ref
Pesticides (*n* = 730)					
	LC amenable pesticides	730	5 µg/kg for 698 cpds 20 µg/kg for 26 cpds Failed for 6 cpds	10 (default MRL)	[21]
Mycotoxins (*n* = 51)					
	Aflatoxins	4	1	2 (B1), Σ4	[18,19]
	Ochratoxin A	1	2	3	
	Zearalenone	1	10	50	
	Deoxynivalenol	1	10	400	
	Fumonisin B1 + B2	2	100	Σ800	
	T-2 HT-2	2	10	Σ25	
	Others (metabolite, emerging, etc.)	23	10–100	-	
	Patulin	1	Failed	10	
	*Alternaria* toxins (except Tenuazonic acid)	4	10	5–10	
	Ergot alkaloids	12	1	Σ 100	
Plant Toxins (*n* = 79)					
	Tropane alkaloids	19	1	Σ 5 (sum of atropine and scopolamine)	[18]
	Pyrrolizidine alkaloids	56	1	Σ 100 (sum of 35 PAs)	
	Others	4	10	-	

## Data Availability

The original contributions presented in the study are included in the article/Supplementary Material, further inquiries can be directed to the corresponding author.

## Supplementary Materials

The following supporting information can be downloaded at: https://www.mdpi.com/article/10.3390/foods13193040/s1, Supplementary Data S1: List of compounds monitored; Supplementary Data S2: Standard solutions’ preparation; Supplementary Data S3: Compound database; Supplementary Data S4: Specific integration parameters; Supplementary Data S5: Validation data.
